# Factors Associated with Willingness to Use Online Medical Consultations Among Survey Participants in China: Insights from the UTAUT Model

**DOI:** 10.3390/healthcare14203401

**Published:** 2026-10-12

**Authors:** Yuqing Wang, Tian Guo, Wenxian Liu, Xiaoling Peng, Shaofan Chen

**Affiliations:** 1 Laboratory for Digital Intelligence & Health Governance, Nanjing Medical University, Nanjing 211166, China; yuqing.wang@stu.njmu.edu.cn (Y.W.); liuwenxian29@163.com (W.L.); xlpeng@njmu.edu.cn (X.P.); 2 School of Health Policy and Management, Nanjing Medical University, No. 101 Longmian Avenue, Nanjing 211166, China; 3 Xuzhou Central Hospital, No. 199, Jiefang South Road, Xuzhou 221009, China; tianguo@stu.njmu.edu.cn

**Keywords:** online medical consultations, UTAUT model, facilitating conditions, logistic regression analysis

## Abstract

**Background:** Online medical consultation services may improve access to healthcare, particularly for patients with chronic diseases. This study aimed to identify factors associated with participants’ willingness to use online medical consultation services based on the Unified Theory of Acceptance and Use of Technology (UTAUT). **Methods:** A cross-sectional study was conducted among 238 participants. Demographic and health-related characteristics were compared using Pearson’s chi-square or Fisher’s exact tests. Educational categories with very small frequencies were combined into broader groups where theoretically appropriate. Mann–Whitney U tests compared composite scores for performance expectancy, effort expectancy, social influence, facilitating conditions, and individual innovativeness. Hierarchical multivariable binary logistic regression was performed to identify factors independently associated with willingness. Model fit and discrimination were assessed using likelihood-ratio tests, pseudo-R^2^ statistics, and ROC-AUC. **Results:** Among 238 participants, 198 (83.2%) were willing to use online medical consultation services. Significant differences in willingness were observed by sex, age group, residential area, and chronic disease history. Participants willing to use online medical consultation services had higher scores for all four UTAUT constructs and individual innovativeness (all *p* < 0.001). In the fully adjusted model, chronic disease history, effort expectancy, and facilitating conditions remained independently associated with willingness. Performance expectancy and social influence were not significant, while individual innovativeness showed a borderline association (*p* = 0.055). The final model demonstrated excellent discrimination (AUC = 0.981, 95% CI: 0.967–0.995). **Conclusions:** Chronic disease history, effort expectancy, and facilitating conditions were key factors associated with willingness to use online medical consultation services. Improving platform usability and strengthening facilitating conditions may support participants’ willingness to use online medical consultation services.

## 1. Introduction

Online medical consultation services, which refer to a new type of healthcare model that integrates traditional medical services with internet-based technologies [1], have emerged as a significant means of accessing healthcare resources in China. With the rapid economic development and growing health awareness among residents, the COVID-19 pandemic, particularly from 2020 onwards, underscored the advantages of these services, resulting in a remarkable surge in usage [2,3]. For instance, as of June 2020, 29.4% of internet users in China used online medical consultation services [4], with some third-party platforms experiencing over a twentyfold increase in consultation volumes. However, despite this rapid growth during the pandemic, the overall usage rate remains relatively low: by the end of 2023, the internet penetration rate reached 77.5%, yet only 38.2% of internet users had accessed online medical consultation services [5]. This discrepancy highlights the necessity for research into the factors influencing participants’ engagement with these services, particularly in the post-pandemic context.

Research consistently identifies perceived usefulness, ease of use, and health consciousness as key drivers of mobile health application adaptation, particularly among specific populations like doctors and college students [6,7,8,9]. Within this framework, studies show online medical consultation can potentially reduce reliance on in-person healthcare, improve patient-provider communication, and ultimately lower costs while enhancing satisfaction [10,11]. Evidence from China and the Mayo Clinic supports these benefits, highlighting reduced strain on healthcare systems [12,13]. Despite these advantages, barriers to widespread adoption persist, particularly in contexts like Australia, where remote psychiatry remains underutilized due to diagnostic challenges from limited patient histories in some online consultation models [14,15]. Furthermore, the existing literature has relatively limited evidence regarding specific services such as online consultations, leaving key factors influencing user willingness underexplored. The Unified Theory of Acceptance and Use of Technology (UTAUT) provides a relevant framework to assess the adoption of telemedicine and health applications, but its applicability to online medical consultation services in China warrants further investigation. Previous studies have mainly focused on healthcare professionals’ acceptance of online consultations, physicians’ perceived barriers, or specific patient concerns such as privacy and information sharing. However, determinants of potential users’ willingness to use online medical consultation services remain relatively underexplored, particularly in the Chinese context. This study extends prior research by applying the UTAUT framework and integrating technology acceptance factors with individual innovativeness and health-related characteristics to examine factors associated with willingness to use online medical consultation services among potential users.

Based on relevant policies and scholarly research, this study defines online medical consultation services as the processes through which users with medical needs communicate with healthcare professionals online via mobile devices—such as smartphones and tablets—utilizing text, voice, and video formats to obtain health guidance, disease prevention advice, and medication recommendations. The current study aims to investigate factors associated with participants’ willingness to use online medical consultation services among the study population in China. By examining demographic, health-related, and technology-acceptance factors, this study seeks to provide evidence that may assist healthcare institutions in optimizing online consultation services and supporting participants’ willingness to use these services. Additionally, it aims to offer insights for policymakers to develop strategies that promote the widespread adoption of online medical consultation services and address the challenges encountered in this domain.

## 2. Methods

### 2.1. Unified Theory of Acceptance and Use of Technology (UTAUT) Model

In the 1980s, Davis proposed the Technology Acceptance Model (TAM) [16], which Venkatesh and colleagues later expanded by integrating eight prominent theories of technology acceptance and use into the UTAUT [17]. The UTAUT model consolidates the core determinants from these theories into four key variables that influence behavioral intention and use behavior: Performance Expectancy (PE), Effort Expectancy (EE), Social Influence (SI), and Facilitating Conditions (FCs). By synthesizing multiple theoretical frameworks, the UTAUT model provides a comprehensive approach to understanding technology acceptance and behavioral intention. When applied to online medical consultation services, the model helps identify factors associated with users’ willingness to use these services, providing theoretical support for understanding their adoption.

### 2.2. Questionnaire Design and Sample Selection

Building upon the UTAUT model, this study formulated a comprehensive framework to investigate the factors associated with participants’ willingness to use online medical consultation services in the context of Internet-based healthcare. The UTAUT model incorporates four principal constructs: effort expectancy, performance expectancy, social influence, and facilitating conditions. While retaining the core variables of the original model, this research extends the framework by incorporating additional factors relevant to the study’s context, including demographic characteristics, individual innovativeness derived from the Diffusion of Innovation theory, and participants’ health status.

Specifically, as for performance expectancy, the current study included the following items: “Using online medical consultation services enables me to access healthcare services more quickly” (PE1), “Using online medical consultation services enhances my effectiveness in addressing health issues” (PE2), and “Using online medical consultation services helps me manage my health” (PE3). Regarding effort expectancy, the study incorporated the items: “Learning to operate online medical consultation services is easy for me” (EE1) and “I believe that online medical consultation services are easy to use” (EE2). As for social influence, the questions included: “People who are important to me, such as family and friends, think that I should use online medical consultation services” (SI1), “People who influence my behavior, such as doctors and specialists, think that I should use online medical consultation services” (SI2), and “Healthcare institutions have supported my use of online medical consultation services” (SI3). For facilitating conditions, the study included: “I have the necessary resources (e.g., devices and internet access) to use online medical consultation services” (FC1) and “Professional personnel or channels are available for assistance when I encounter difficulties with online medical consultation services” (FC2). Finally, with respect to individual innovativeness, the study included the items: “I am curious about many things” (II1), “I find trying new information technologies interesting” (II2), and “I am usually one of the first among my peers to try new information products” (II3). All UTAUT and individual innovativeness items were assessed using a five-point Likert scale ranging from 1 (Strongly Disagree) to 5 (Strongly Agree).

The internal consistency and sampling adequacy of the questionnaire were assessed using Cronbach’s α, the KMO measure, and Bartlett’s test of sphericity. The overall Cronbach’s α coefficient was 0.867, indicating good internal consistency. The KMO value was 0.923, and Bartlett’s test was significant (*p* < 0.05), indicating that the data were suitable for factor analysis.

To ensure data completeness and quality, stringent inclusion and exclusion criteria were applied. The inclusion criteria were as follows: (1) Participants capable of independently completing the questionnaire; (2) Adults aged 18 and above, or minors with parental consent; (3) Complete responses to all questions; (4) Consent to participate and commitment to providing honest responses. The exclusion criteria were as follows: (1) Questionnaires completed in less than one minute, indicating potential inattentiveness; (2) Responses displaying highly repetitive answers across multiple items, indicating careless answering.

A non-probability convenience sampling approach was used to recruit participants. The survey was conducted between March and April 2024 through multiple online platforms, with a primary focus on the general population. A total of 267 responses were initially collected. After applying the predefined exclusion criteria, 238 valid responses were retained, yielding a valid response rate of 89.1%. The questionnaire was disseminated through multiple online channels, including WeChat, Weibo, online forums, and Wenjuanxing, to reach participants with diverse demographic characteristics, including different age groups, occupations, and educational levels. Participants voluntarily completed the questionnaire. All submitted questionnaires were manually reviewed, and responses were excluded if the completion time was less than 1 min, if responses were incomplete, or if response patterns indicated repetitive or invalid answering. These procedures were implemented to ensure the completeness and quality of the final dataset.

### 2.3. Statistical Methods

Willingness to use online medical consultation services was measured using a single categorical item with two response options: “Willing” and “Unwilling.” The outcome variable was directly coded as 1 = willing and 0 = unwilling and was not derived by dichotomizing a Likert-scale intention score.

To examine differences in participants’ willingness to use online medical consultation services, categorical variables were compared using Pearson’s chi-square test for sex, residential area, and chronic disease history. Fisher’s exact test was used for age group, educational level, and household monthly income when expected cell frequencies were low.

For the UTAUT-related constructs, item-level responses were aggregated by calculating composite mean scores for performance expectancy (PE1–PE3), effort expectancy (EE1–EE2), social influence (SI1–SI3), and facilitating conditions (FC1–FC2). Individual innovativeness was also calculated as a composite mean score based on its corresponding measurement items. The Mann–Whitney U test was used to compare the distributions of these five composite scores between participants who were willing and unwilling to use online medical consultation services.

The univariate analyses were considered exploratory rather than confirmatory and were used to describe preliminary group differences. Therefore, no formal adjustment for multiple comparisons was applied, and the resulting *p*-values were interpreted cautiously. Variables were not selected for inclusion in the multivariable models solely on the basis of univariate statistical significance.

To assess potential common method bias, Harman’s single-factor test was conducted. All scale items were entered into an unrotated exploratory factor analysis, and the proportion of total variance explained by the first factor was examined. A first-factor variance below 50% was considered to indicate that a single factor did not account for the majority of the variance.

To identify factors associated with participants’ willingness to use online medical consultation services, theory-driven multivariable binary logistic regression was performed because the dependent variable was dichotomous. The outcome variable was coded as 1 = willing and 0 = unwilling. The analytical procedure followed a prespecified hierarchical sequence: demographic and health-related variables were entered in Model 1, the four UTAUT constructs were added in Model 2, and individual innovativeness was added in Model 3. Model comparisons were then performed using likelihood-ratio tests and model-fit statistics.

Categorical predictors were entered using dummy coding. The reference categories were female for sex, 60 years or older for age, bachelor’s degree or above for educational level, urban residence for residential area, monthly household income above 8000 RMB for household income, and presence of chronic disease for chronic disease history.

The logistic regression formula was specified as follows:

$$\log\frac{P}{1-P}=\beta_{0}+\beta_{1}X_{1}+\beta_{2}X_{2}+\beta_{n}X_{n}$$

where P represents the probability of willingness to use online medical consultation services, 1−P represents the probability of unwillingness, $\beta_{0}$ is the intercept, and $\beta_{1}$ represents the regression coefficient for each independent variable $X_{i}$. Accordingly, a positive coefficient corresponds to higher odds of willingness, whereas a negative coefficient corresponds to lower odds of willingness. Odds ratios (OR) with 95% confidence intervals (CI) were reported to quantify the associations between the explanatory variables and willingness to use online medical consultation services.

Multicollinearity among the independent variables was assessed using the variance inflation factor (VIF), with VIF values < 10 indicating no serious multicollinearity. Likelihood ratio tests were used to compare the nested models and assess whether the sequential addition of variables introduced at each modeling step. Model fit was evaluated using the −2 log-likelihood, Cox–Snell pseudo-R^2^, and Nagelkerke pseudo-R^2^. The discriminative performance of the logistic regression models to distinguish between willingness and unwillingness was assessed using the area under the receiver operating characteristic curve (ROC-AUC). All statistical tests were two-sided, and a *p*-value < 0.05 was considered statistically significant.

### 2.4. Ethics Consideration

The current study adhered to established ethical guidelines for research involving human subjects. Informed consent was obtained from all participants, who were thoroughly briefed on the study’s purpose, the voluntary nature of their participation, and their right to withdraw at any time. To ensure confidentiality, no personal identifiers were collected, and all responses were anonymized. Data were securely stored and made accessible only to the research team. The survey posed minimal risk to participants, as it solely explored perceptions of online medical consultation services. Ethical approval was obtained from Nanjing Medical University, ensuring adherence to ethical standards throughout the research process.

## 3. Results

### 3.1. Demographic Characteristics of Participants

Table 1 shows the demographic characteristics of the participants. The majority were female (60.9%), and 58.8% were aged 18–35 years. In terms of educational level, 68.1% of the respondents held a bachelor’s degree. Geographically, 56.7% resided in urban areas, while 43.3% lived in rural areas. Regarding household monthly income, 37.8% reported RMB 4000–8000, 27.3% reported more than RMB 8000, 24.4% reported less than RMB 4000, and 10.5% did not disclose their income. Additionally, 52.9% reported a history of chronic disease, while others did not.

### 3.2. Univariate Analysis

Among the 238 participants, 198 (83.2%) were willing to use online medical consultation services, while 40 (16.8%) were unwilling. Six demographic and health-related variables, including sex, age group, educational level, residential area, household monthly income and chronic disease history, were included for univariate comparison.

As shown in Table 2, significant differences in willingness to use online medical consultation services were observed by sex (*p* = 0.007), age group (*p* < 0.001), residential area (*p* = 0.001), and chronic disease history *(p* < 0.001). In contrast, educational level and household monthly income did not differ significantly between the two groups (both *p* > 0.05).

As shown in Table 3, participants who were willing to use online medical consultation services had significantly higher median scores for performance expectancy, effort expectancy, social influence, facilitating conditions, and individual innovativeness than those who were unwilling to use the services (all *p* < 0.001).

Harman’s single-factor test showed that the first factor accounted for 40.586% of the total variance, which was below the commonly used 50% threshold. This finding suggests that no single factor accounted for the majority of the variance and that substantial common method bias was unlikely to have materially affected the results.

Overall, the univariate analyses indicated significant group differences across several demographic, health-related, and technology-related variables. Although educational level and household monthly income were not significant in the univariate analyses, they were retained as covariates in the multivariable models based on theoretical considerations.

### 3.3. Hierarchical Multivariable Logistic Regression

A hierarchical multivariable binary logistic regression analysis was performed to identify factors independently associated with willingness to use online medical consultation services and to assess the incremental contribution of UTAUT-related constructs and individual innovativeness after adjustment for demographic and health-related factors.

As presented in Table 4, three nested models were constructed using a theory-driven hierarchical approach. Model 1 included demographic and health-related covariates. Model 2 additionally incorporated four UTAUT constructs, namely performance expectancy, effort expectancy, social influence, and facilitating conditions. Model 3 further included individual innovativeness.

Of note, the under-18 subgroup contained only eight participants (1 willing and 7 unwilling). After the UTAUT constructs were introduced, the estimates for this subgroup became highly unstable, as reflected by the extremely wide confidence intervals in Models 2 and 3. These estimates were therefore not interpreted substantively. In Model 1, sex, age group, residential area, and chronic disease history were significantly associated with willingness to use online medical consultation services. After the four UTAUT constructs were added in Model 2, the associations of sex, age group, and residential area with willingness became non-significant, whereas chronic disease history remained associated with willingness. Among the UTAUT constructs, effort expectancy and facilitating conditions were positively associated with willingness to use online medical consultation services, while performance expectancy and social influence were not statistically significant.

In the fully adjusted Model 3, chronic disease history, effort expectancy, and facilitating conditions remained significantly associated with willingness. Individual innovativeness showed a borderline association with willingness (*p* = 0.055); its independent association did not reach statistical significance and was therefore interpreted with caution. The logistic regression equation for the final model is

$$log\frac{P}{1-P}=-20.910-3.308*X_{chronic}+1.453*X_{EE}+1.467X_{FC}$$

where *P* is the probability of willingness to use online medical consultation services, 1 − *P* is the probability of unwillingness. *X_chronic_* represents the absence of a chronic disease history, *X_EE_* represents effort expectancy, and *X_FC_* represents facilitating conditions. The outcome variable was coded as 1 for willingness to use online medical consultation services and 0 for unwillingness. Therefore, odds ratios greater than 1 indicate higher odds of willingness to use online medical consultation services.

Diagnostic analyses indicated no serious multicollinearity among the predictors, with all VIF values below 10. Likelihood-ratio tests were used to assess whether the sequential addition of variable blocks improved model fit. Model performance was further evaluated using the −2 log-likelihood statistic, Nagelkerke pseudo-R^2^, and the receiver operating characteristic (ROC) curve. These indices collectively indicated satisfactory model fit and discriminative performance.

**Figure 1:**
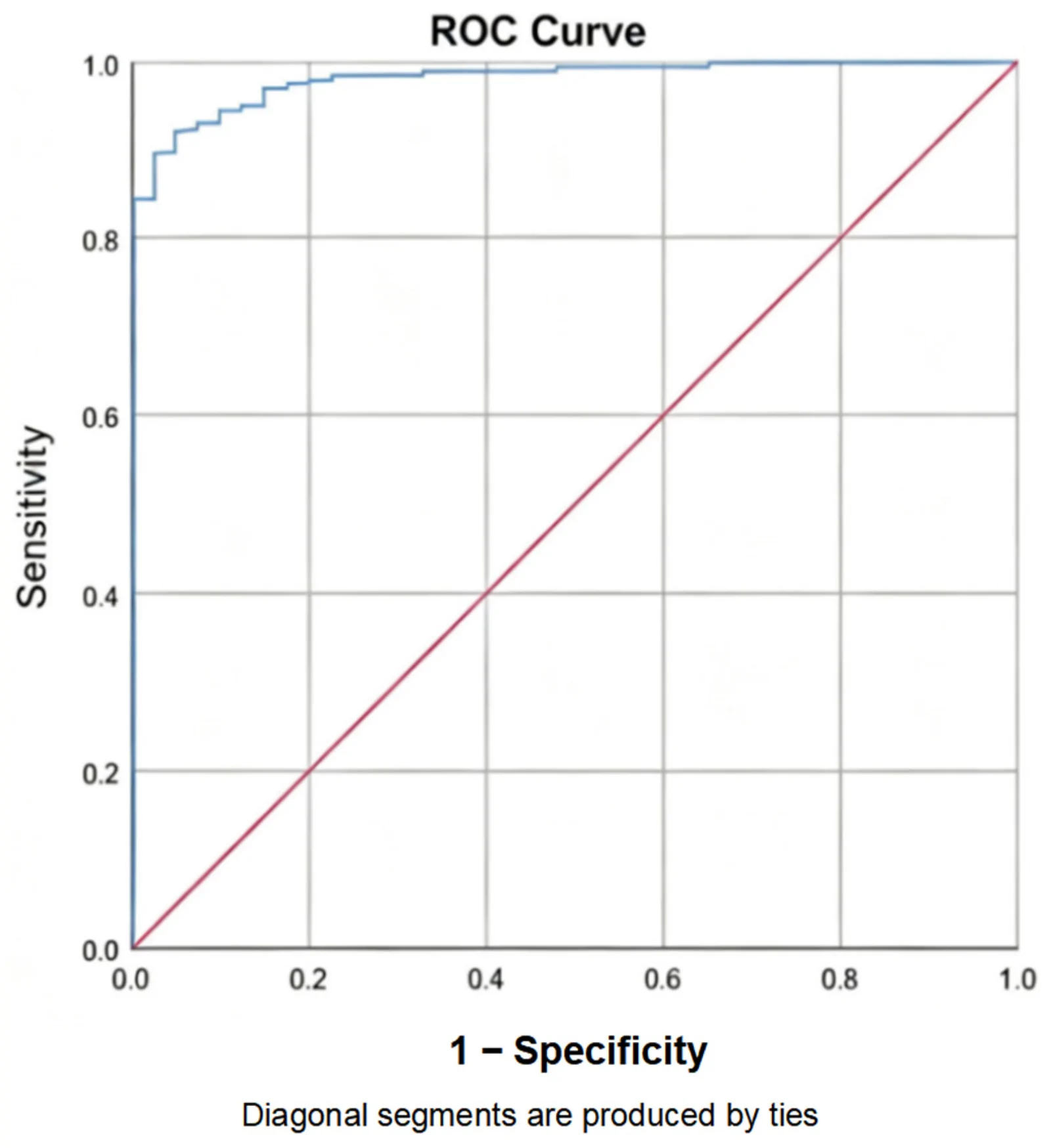
Receiver operating characteristic (ROC) curve of the hierarchical multivariable logistic regression model. The blue stepped curve represents the ROC curve of the prediction model, plotting sensitivity (true positive rate) against 1 − Specificity (false positive rate) across all classification thresholds. The red diagonal line represents the reference line for chance performance, corresponding to a random classifier with an area under the curve (AUC) of 0.5. Diagonal segments in the blue curve are produced by ties in predicted scores.

The ROC curve is shown in Figure 1. It was generated to assess the discriminative performance of the final logistic regression model. The area under the curve (AUC) was 0.981 (95% CI: 0.967–0.995; *p* < 0.001), indicating excellent discrimination between participants who were willing and unwilling to use online medical consultation services. At the default probability cutoff of 0.5, the final model correctly classified 191 of 198 willing participants and 35 of 40 unwilling participants, with 7 willing participants and 5 unwilling participants misclassified. The apparent sensitivity, specificity, and overall classification accuracy were 96.5%, 87.5%, and 95.0%, respectively. Given the relatively small sample size and the limited number of participants in the unwilling group, however, the observed discrimination should be interpreted cautiously and requires validation in larger independent samples.

## 4. Discussion

In the current study, we used the UTAUT model to identify key factors associated with participants’ willingness to use online medical consultation services. In the fully adjusted model, chronic disease history, effort expectancy, and facilitating conditions remained significantly associated with willingness to use online medical consultation services. Specifically, higher effort expectancy (OR = 4.28, 95% CI: 1.20–15.26, *p* = 0.025) and facilitating conditions (OR = 4.33, 95% CI: 1.14–16.46, *p* = 0.031) were associated with higher odds of willingness. In contrast, performance expectancy and social influence were not statistically significant after adjustment, while individual innovativeness showed only a borderline association (OR = 3.62, 95% CI: 0.97–13.42, *p* = 0.055). These findings suggest that perceived ease of use and external support may have more robust associations with willingness than perceived usefulness, social influence, or innovativeness in the present sample. These findings are broadly consistent with Garavand et al. (2019) [18], who applied the UTAUT model to examine adoption among medical science students in mobile health applications. Their study revealed that while factors such as effort expectancy and facilitating conditions were significantly associated with technology use, performance expectancy and social influence showed no direct impact on behavioral intention. Unlike previous studies focusing primarily on healthcare professionals’ acceptance or specific barriers such as privacy concerns, this study examined potential users’ willingness to use online medical consultation services and incorporated technology acceptance, individual innovativeness, and health-related characteristics within a unified framework.

A history of chronic disease was significantly associated with participants’ willingness to use online medical consultation services. Individuals with chronic conditions may be more inclined to use these services because of their frequent healthcare needs and the potential convenience of remote consultations, which may reduce the time and frequency of in-person visits. This association may therefore reflect the greater need for continuous healthcare access among individuals with chronic conditions. Previous evidence has highlighted telemedicine as an increasingly relevant approach for providing remote healthcare for patients with chronic diseases and has examined patients’ willingness to pay for such services [19]. These findings provide contextual support for the observed association between chronic disease history and willingness to use online medical consultation services in the present study.

Effort expectancy and facilitating conditions were also significantly associated with participants’ willingness to use online medical consultation services. Participants who perceived these services as easier to learn and use were more willing to use them. This may be because simple and convenient operating procedures can reduce the technical burden associated with online consultation and increase participants’ confidence in using the service. Similarly, Rahi (2022) [20] found that effort expectancy significantly influenced the adoption of telemedicine applications, highlighting the relevance of perceived ease of use in digital healthcare adoption. Facilitating conditions were also positively associated with willingness to use online medical consultation services. When participants have access to appropriate resources, technical support, and necessary assistance, difficulties encountered during online consultation may be reduced and support users’ willingness to use these services. Alkhalifah (2022) [21] similarly found that facilitating conditions significantly influenced patient acceptance of mobile health, supporting the importance of adequate external support in digital healthcare adoption. In contrast, although performance expectancy and social influence were significantly associated with willingness in the univariate analysis, neither remained significant after adjustment for other variables. This finding suggests that their associations may be partly explained by other factors included in the model. 

Individual innovativeness was significantly associated with willingness to use online medical consultation services in the univariate analysis, but this association did not reach statistical significance in the final model (*p* = 0.055). This finding suggests that participants who are more open to trying new technologies may initially show greater willingness to use online medical consultation services; however, individual innovativeness may have a limited independent contribution after other technology-related and health-related factors are considered. Park and Kim (2010) [22] reported that individual innovativeness was associated with the acceptance of IT-based innovations in healthcare settings.

The difference between the present findings and previous research may be related to differences in study populations, healthcare technologies, and other factors included in the analysis. While this study offers valuable insights, several limitations should be acknowledged. First, the online recruitment approach and the characteristics of the study population may have introduced selection and digital-access biases, limiting the representativeness and generalizability of the findings to the broader Chinese population. Second, the cross-sectional design does not allow causal relationships or changes in willingness over time to be established. Longitudinal studies are therefore needed to examine how willingness to use online medical consultation services may evolve over time.

Third, the outcome was imbalanced, with only 40 participants reporting unwillingness to use online medical consultation services. This imbalance may compromise the precision and stability of logistic regression coefficient estimates, particularly for less frequent covariate subgroups. No formal a priori sample-size calculation was conducted. Given the relatively small number of participants reporting unwillingness, the regression analysis was considered exploratory and was primarily intended to examine associations rather than to develop a definitive prediction model. In addition, because the ROC-AUC was calculated using the same sample in which the model was fitted, the observed AUC represents apparent discrimination and may overestimate out-of-sample performance. Therefore, the regression findings should be interpreted cautiously and confirmed in larger and more diverse samples.

Finally, the study did not include some healthcare-specific factors that may be relevant to willingness to use online medical consultation services, such as privacy concerns, perceived risk, trust, and service quality. Future research incorporating these factors, together with larger samples and longitudinal designs, may provide a more comprehensive understanding of willingness to use online medical consultation services. Future studies may further examine actual utilization behavior rather than willingness alone through longitudinal follow-up designs.

## 5. Conclusions

This cross-sectional study applied the UTAUT framework to examine factors associated with willingness to use online medical consultation services among 238 survey participants in China. In the fully adjusted hierarchical logistic regression model, chronic disease history, effort expectancy, and facilitating conditions remained significantly associated with willingness, whereas performance expectancy and social influence were not statistically significant, and individual innovativeness showed a borderline association. These findings highlight the potential importance of perceived ease of use and facilitating conditions in willingness to use online medical consultation services. Improving the usability and accessibility of online medical consultation platforms and providing adequate technical support and feedback mechanisms may help support participants’ willingness to use these services. Given the cross-sectional design and convenience sampling approach, these findings should be interpreted as associations and confirmed in larger and more representative samples.

## Figures and Tables

**Table 1:** Demographic Characteristics of Participants.

Variable	Frequency (*n*)	Percentage (%)
Sex		
male	93	39.1
female	145	60.9
Age		
Under 18	8	3.4
18–35	140	58.8
36–59	70	29.4
60 and above	20	8.4
Educational level		
Junior High or below	19	8.0
Secondary School	36	15.1
Bachelor	162	68.1
Graduate or above	21	8.8
Residential area		
Rural	103	43.3
Urban	135	56.7
Household Monthly Income		
Below 4000 RMB	58	24.4
4000–8000 RMB	90	37.8
Above 8000 RMB	65	27.3
Prefer not to say	25	10.5
Chronic Disease History		
No	112	47.1
Yes	126	52.9

**Table 2:** Chi-square test.

Variable	Willingness to Use (*n*%)		Total (*n*)	Chi-Square	*p* Value
	Unwilling	Willing			
Total	40 (16.8%)	198 (83.2%)	238		
Sex					
Male	8 (8.6%)	85 (91.4%)	93	7.349	0.007 *
Female	32 (22.1%)	113 (77.9%)	145		
Age					
Under 18	7 (87.5%)	1 (12.5%)	8		<0.001 **
18–35	24 (17.1%)	116 (82.9%)	140		
36–59	7 (10.0%)	63 (90.0%)	70		
60 and above	2 (10.0%)	18 (90.0%)	20		
Educational level					
Junior High or below	1 (5.3%)	18 (94.7%)	19		0.181
Secondary School	9 (25.0%)	27 (75.0%)	36		
Bachelor or above	30 (16.4%)	132 (83.6%)	183		
Residential area					
Rural	8 (7.8%)	95 (92.2%)	103	10.613	0.001 **
Urban	32 (23.7%)	103 (76.3%)	135		
Household Monthly Income					
Below 4000 RMB	14 (24.1%)	44 (75.9%)	58		0.406
4000–8000 RMB	12 (13.3%)	78 (86.7%)	90		
Above 8000 RMB	14 (21.5%)	51 (78.5%)	65		
Prefer not to say	0 (0.0%)	25 (100.0%)	25		
Chronic Disease History					
Yes	7 (5.6%)	119 (94.4%)	126	24.24	<0.001 **
No	33 (29.5%)	79 (70.5%)	112		

* *p* < 0.05, ** *p* < 0.001.

**Table 3:** Mann–Whitney U Test.

Variable	UnwillingM (P25, P75)	WillingM (P25, P75)	z	*p* Value
Performance Expectancy	3.00 (2.67, 3.67)	4.33 (3.92, 4.33)	−7.58	<0.001 **
Effort Expectancy	3.50 (2.50, 3.50)	4.50 (4.00, 4.50)	−7.70	<0.001 **
Social Influence	3.00 (2.00, 3.59)	4.33 (4.00, 4.33)	−8.06	<0.001 **
Facilitating Conditions	3.00 (2.50, 3.88)	4.50 (4.00, 4.50)	−7.45	<0.001 **
Individual Innovativeness	3.33 (2.67, 4.00)	4.00 (3.67, 4.33)	−5.40	<0.001 **

** *p* < 0.001.

**Table 4:** Hierarchical Multivariable Binary Logistic Regression.

Variable	Model 1		Model 2		Model 3	
	OR (95% CI)	*p*	OR (95% CI)	*p*	OR (95% CI)	*p*
Sex (Ref: Female)						
Male	3.78 (1.16–12.28)	0.027 *	1.52 (0.26–9.10)	0.644	1.09 (0.17–6.80)	0.929
Age (Ref: 60 and above)						
Under 18	0.01 (0.00–0.22)	0.004 *	44.10 (0.14–13,678.55)	0.196	59.32 (0.01–357,171.46)	0.358
18–35	4.11 (0.59–28.75)	0.154	4.41 (0.15–130.79)	0.390	7.08 (0.20–251.72)	0.283
36–59	4.28 (0.57–31.97)	0.156	3.41 (0.13–89.44)	0.461	6.43 (0.20–212.62)	0.297
Educational level (Ref: Bachelor or above)						
Junior High or below	1.74 (0.18–16.67)	0.631	2.44 (0.09–68.17)	0.599	2.70 (0.05–147.98)	0.627
Secondary School	1.03 (0.18–6.00)	0.971	0.73 (0.05–10.73)	0.820	1.63 (0.08–35.59)	0.755
Residential area (Ref: Urban)						
Rural	4.90 (1.70–14.09)	0.003 *	2.79 (0.53–14.63)	0.225	2.33 (0.39–13.78)	0.352
Household Monthly Income (Ref: Above 8000 RMB)						
Below 4000 RMB	1.05 (0.35–3.13)	0.930	1.27 (0.20–7.97)	0.796	1.22 (0.20–7.49)	0.831
4000–8000 RMB	0.91 (0.34–2.43)	0.848	1.11 (0.25–4.99)	0.890	1.87 (0.34–10.15)	0.469
Chronic Disease History (Ref: Yes)						
No	0.08 (0.02–0.30)	<0.001 **	0.04 (0.00–0.47)	0.010 *	0.04 (0.00–0.45)	0.010 *
Performance Expectancy	—	—	3.73 (0.83–16.72)	0.085	2.62 (0.55–12.58)	0.228
Effort Expectancy	—	—	4.72 (1.41–15.79)	0.012 *	4.28 (1.20–15.26)	0.025 *
Social Influence	—	—	1.92 (0.67–5.51)	0.225	2.33 (0.76–7.14)	0.140
Facilitating Conditions	—	—	4.98 (1.31–19.00)	0.019 *	4.33 (1.14–16.46)	0.031 *
Individual Innovativeness	—	—	—	—	3.62 (0.97–13.42)	0.055

Ref, reference category. OR, odds ratio; CI, confidence interval. —, variable not included in the model. * *p* < 0.05, ** *p* < 0.001. The under-18 subgroup included only eight participants (1 willing and 7 unwilling). The extremely wide confidence intervals for this subgroup in Models 2 and 3 reflect sparse data and unstable estimation consistent with quasi-complete separation; these estimates should therefore be interpreted with particular caution.

## Data Availability

The data are not publicly available due to privacy and ethical restrictions.
